# Cellular crosstalk of mesangial cells and tubular epithelial cells in diabetic kidney disease

**DOI:** 10.1186/s12964-023-01323-w

**Published:** 2023-10-16

**Authors:** Shan Jiang, Hua Su

**Affiliations:** Department of Nephrology, Union Hospital, Tongji Medical College, Huazhong University of Science and Technology, Wuhan, 430022 China

**Keywords:** Diabetic kidney disease, Cellular crosstalk, Mesangial cells, Tubular epithelial cells, High glucose

## Abstract

Diabetic kidney disease (DKD) is a major cause of end-stage renal disease and imposes a heavy global economic burden; however, little is known about its complicated pathophysiology. Investigating the cellular crosstalk involved in DKD is a promising avenue for gaining a better understanding of its pathogenesis. Nonetheless, the cellular crosstalk of podocytes and endothelial cells in DKD is better understood than that of mesangial cells (MCs) and renal tubular epithelial cells (TECs). As the significance of MCs and TECs in DKD pathophysiology has recently become more apparent, we reviewed the existing literature on the cellular crosstalk of MCs and TECs in the context of DKD to acquire a comprehensive understanding of their cellular communication. Insights into the complicated mechanisms underlying the pathophysiology of DKD would improve its early detection, care, and prognosis.

Video Abstract

**Supplementary Information:**

The online version contains supplementary material available at 10.1186/s12964-023-01323-w.

## Background

Diabetic kidney disease (DKD) is a major complication of diabetes, with approximately 30–40% of patients with diabetes developing DKD [1]. Projections indicate a 54% increase in diabetes prevalence in the US by 2030, with a concomitant increase in the occurrence of DKD [2]. DKD is characterized by persistent proteinuria that progresses to end-stage renal disease (ESRD). DKD contributes to approximately 40–50% of ESRD cases, imposing a significant economic burden globally [3, 4].

Effectively addressing the clinical impact of DKD hinges upon a comprehensive understanding of the intricate signaling pathways involved in its pathogenesis. DKD originates from diabetes, with the initial trigger being hyperglycemia (high blood glucose). DKD gradually develops owing to various conditions, including hypertension, glomerular hyperperfusion, hyperlipidemia, hyperaminoacidemia, and other metabolic and hemodynamic abnormalities [5]. High glucose (HG) conditions activate the Janus kinase (JAK)/signal transducer and activator of transcription (STAT) pathway and inhibit autophagy in podocytes [6]. Further, lipotoxicity leads to cellular hypoxia, mitochondrial dysfunction, and macrophage recruitment and activation [7, 8].

Various molecules, including advanced glycation end products (AGEs) and transforming growth factor-β (TGF-β), as well as increased oxidative stress decrease the availability of vasodilatory nitric oxide, thereby aggravating renal hypoxia [9]. AGEs induce endoplasmic reticulum (ER) stress in podocytes, consequently prompting podocyte apoptosis [10]. Interleukin (IL)-1 promotes the release of prostaglandin E2, leading to glomerular hyperperfusion [11]. Furthermore, the TGF-β, mitogen-activated protein kinase, Notch, JAK/STAT, and Wnt/β-catenin pathways promote renal tissue fibrosis in DKD [12].

Intercellular crosstalk plays a crucial role in DKD pathophysiology and has recently gained prominence as a research topic. For example, studies have reported cellular crosstalk between endothelial cells (ECs) and podocytes [13–16]. Moreover, Dong et al. demonstrated the mechanisms underlying the crosstalk of glomerular cells in DKD, providing a direction for future research [17]. Most studies typically focus on ECs and podocytes, both of which play important roles in DKD development. However, it is imperative to underscore the potential role of mesangial cells (MCs) and renal tubular epithelial cells (TECs) in DKD. Regarding DKD, the crosstalk of TECs and MCs with other cells has been demonstrated in several studies. For example, Hartner et al. demonstrated the crucial role of integrin α8 expression on MCs, highlighting its protective effect on podocytes [18]. Moreover, tubular-derived IL-1β and monocyte chemoattractant protein-1 (MCP-1) recruit macrophages to promote inflammation [19, 20]. This review supplements the existing literature on cellular crosstalk by focusing on the crosstalk of TECs and MCs with other cells in the context of DKD. This comprehensive understanding of the pathogenesis of DKD and its unique biological processes, including cellular crosstalk, will enable the identification of potential therapeutic targets.

## Cellular crosstalk of MCs

Owing to their contractility, MCs play an important role in maintaining capillary structure and regulating glomerular filtration [21]. Moreover, activated MCs function in phagocytosis and antigen presentation [22, 23]. White et al. [24] reported that the number of renal MCs increases in patients with diabetes compared with that in non-diabetic controls. With disease progression, the number of MCs decreases, and the mesangial matrix expands [25]. Suppression of the Wnt/β-catenin pathway and the generation of Ras/Rac1-dependent superoxide under an HG environment are associated with the destruction of MCs [26]. An HG environment causes ferroptosis in MCs via the high-mobility group box-1/nuclear factor E2-related factor 2 pathway [27]. Elevated glomerular perfusion pressure enhances the mechanical forces acting on the MCs and promotes mesangial matrix production [28]. Excessive production and expansion of the mesangial matrix are crucial processes in the development of glomerulosclerosis in DKD. Finally, the formation of characteristic Kimmelstiel–Wilson nodules indicates an advanced disease and poor prognosis [29]. The decreased number of MCs induces glomerular basement membrane (GBM) damage, cluster collapse, and aneurysm [30]. Owing to the significance of MCs in DKD progression, their processes in cellular crosstalk are of great interest. The current state of research on the crosstalk of MCs with other cells is described in detail below.

### Cellular crosstalk of MCs with podocytes

#### TGF-β1 in exosomes

TGF-β, a key pathogenic factor in DKD, promotes the accumulation of extracellular matrix (ECM) and fibrosis [31, 32]. TGF-β1 is the most important isoform of TGF-β. Using microarray analysis, Liu et al. [33] showed that TGF-β1 was one of the differentially expressed genes in early diabetic nephropathy (DN) and non-diabetic samples as its expression was upregulated. Further, serum TGF-β1 has been identified as a risk factor for developing DN and may even serve as a potential biomarker, as shown in a meta-analysis conducted by Mou et al. [34]. However, clinical trials have not demonstrated the effectiveness of anti-TGF-β1 antibodies in preventing DKD progression, suggesting a complex role of TGF-β1 in DKD [35]. Therefore, additional investigations into the mechanisms underlying TGF-β1 activity in DKD, including its involvement in cellular crosstalk, are required.

By culturing MCs under HG conditions and analyzing the expression of exosomal markers (CD63 and TSG101), Wang et al. [36] demonstrated that MCs secreted exosomes. Furthermore, they incubated PHK67-labeled exosomes with podocytes and reported that exosomes were taken up by podocytes, resulting in a decreased expression of nephrin, podocin, and WT-1. In addition, by co-culturing MCs with podocytes in a Transwell, they demonstrated the crosstalk between MCs and podocytes, which plays a pathogenic role in inducing apoptosis and inhibiting cell adhesion under HG conditions. Wang et al. [36] found that HG-treated MCs secreted exosomes with increased TGF-β1 expression, targeting the phosphatidylinositol 3-kinase (PI3K)/AKT pathway in podocytes. TGF-β1 reduction in MCs reduced the podocyte damage induced by exosomes from HG-treated MCs. In conclusion, TGF-β1 from MCs triggers the PI3K/AKT pathway in podocytes via exosomes, leading to podocyte apoptosis [36].

#### ER-associated degradation

ER-associated degradation (ERAD), in addition to the unfolded protein response (UPR) and macroautophagy, is an important mechanism for maintaining ER homeostasis. The ER is essential for protein synthesis, folding, and maturation in eukaryotic cells; however, disruption of its homeostasis results in the accumulation of unfolded or misfolded proteins, leading to ER stress [37]. ER stress is widely documented in patients with DKD. For example, Morse et al. [38] demonstrated an upregulation in C/EBP homologous protein (CHOP) expression in the kidneys of diabetic mice, which increased the expression of tribbles homolog 3 (TRB3), an ER stress-related protein. Moreover, TRB3 inhibited MCP-1 expression and mitigated inflammatory damage. Borsting et al. [39] reported that TRB3-knockout diabetic mice experienced more severe ER stress and proteinuria than that in wild-type (WT) mice.

Fujimoto et al. [40] observed an increased B-cell lymphoma 2 (Bcl-2) associated protein X (Bax)/Bcl-2 ratio and CHOP expression in podocytes exposed to the culture supernatant of HG-treated MCs. Moreover, the levels of phosphorylated inositol-requiring transmembrane kinase/endoribonuclease 1α, Derlin-1, Derlin-2 (an ERAD-related protein), and nephrin were decreased in podocytes. These findings indicate that the culture supernatant of HG-treated MCs promoted apoptosis and inhibited ERAD in podocytes. However, the mechanism underlying this phenomenon remains unclear and requires further investigation. Generally, molecules such as ER degradation-enhancing α-mannosidase-like protein, molecular chaperones, and Yos9 play significant roles in identifying and transporting proteins for ERAD, presenting promising directions for future research [41, 42].

#### Integrin α8

Using transcriptome analysis, Woroniecka et al. [43] demonstrated that integrin signaling pathways are the primary differential regulatory pathways in DKD. Studies have demonstrated the selective expression of integrin α8 in the MCs of the glomerulus in DKD and its relevance to cellular crosstalk [44, 45]. Hartner et al. [18] reported increased integrin α8 expression in DN. The authors induced diabetes using streptozotocin (STZ) in integrin α8-deficient and WT mice. Integrin α8-deficient diabetic mice exhibited more severe podocyte damage, manifesting as reduced expression of podocyte markers, including WT-1, vimentin, and nephrin. This finding demonstrates the protective relationship mediated by integrin α8 between MCs and podocytes in DKD. Although podocyte dedifferentiation may be involved, the exact mechanism through which integrin α8 deficiency in MCs triggers podocyte injury remains unknown.

The study of integrins in exosomes has significantly advanced our understanding of tumor progression and metastasis. Hoshino et al. [46] showed that exosomes expressing integrin α6β4 and αVβ5 are preferentially distributed in lung and liver tissues, respectively. Moreover, exosomes expressing integrin αvβ6 and αvβ3 are involved in the adhesion and migration of prostate cancer cells [47, 48]. The potential for integrins to exert crosstalk effects in DKD via exosomes has garnered research interest.

However, relatively few studies have investigated the role of integrins in DKD. The receptor of AGEs and integrin αvβ3 play a crucial role in initiating the pathogenic signaling mediated by soluble urokinase plasminogen activator receptor in podocytes [49]. In diabetic mice kidneys, integrin α1 deficiency caused severe mesangial expansion and GBM thickening compared to that in WT diabetic mice [50]. Integrin β6 promoted epithelial–mesenchymal transition in renal TECs, thus facilitating DKD progression [51].

Shenaz et al. [52] found that integrin αvβ8 in MCs protects ECs through TGF-β isolation; however, the absence of integrin αvβ8 may cause further TGF-β activation. Altered expression of integrin αvβ8 in DKD may be involved in the crosstalk between MCs and ECs. Although the crosstalk of integrins in exosomes in DKD remains unexplored, some studies provide potential directions for research. The expression of integrin β1 in podocytes cultured under HG conditions was increased compared to that in cells cultured under normal glucose conditions [53]. Meanwhile, the integrin β1/cell surface glucose-regulated protein (GRP) 78 complex has been demonstrated to be involved in the TGF-β1 signaling pathway in MCs to promote fibrosis [54]. Furthermore, GRP78, an important UPR regulator, can translocate to the cell surface under ER stress conditions and is activated to conduct signals [55]. Further studies are required to investigate the potential for the high expression of integrin β1 in podocytes to facilitate crosstalk with MCs via exosomes in DKD. Moreover, Karamessinis et al. [56] reported decreased expression of integrins α3, β1, and α5 in TECs under HG conditions, whereas Jin et al. [57] observed a gradual increase in the expression of integrins α3, β1, and α5 in MCs in the DN. This change in expression patterns across regions, possibly mediated by cellular crosstalk, requires experimental verification.

### Cellular crosstalk of MCs with macrophages

#### MCP-1

Urine MCP-1 level is related to the disease progression in patients with DKD and is a potential biomarker of DKD [58]. Kang et al. [59] reported increased expression of MCP-1 in HG-treated MCs. Yang et al. [60] evaluated the levels of reactive oxygen species (ROS) and nuclear factor κB (NF-κB) pathway-related proteins, such as NF-κB p65 and p-NF-κB p65, and reported that HG promoted MCP-1 production in MCs by activating the ROS/NF-κB pathway. Moreover, according to Chen et al. [61], miR-192 upregulation is an upstream mechanism of HG-induced MCP-1 synthesis in MCs. Researchers have widely acknowledged the importance of MCP-1/C-C chemokine receptor type 2 (CCR2, the MCP-1 receptor) in attracting macrophages and driving inflammatory responses [62]. MCP-1 binds CCR2 to recruit macrophages [63]. Kanamori et al. [64] specifically blocked the MCP-1/CCR2 pathway in diabetic mice using propagermanium, a CCR2 antagonist, and observed reduced macrophage infiltration compared to that in control diabetic mice. Furthermore, Ishibashi et al. [65] suggested that glucagon-like peptide-1 downregulated the expression of MCP-1 produced by AGE-treated MCs and exerted anti-inflammatory effects. These findings support the hypothesis that MCs cultivated in an HG environment can recruit macrophages via MCP-1/CCR2 to enhance inflammatory responses.

Meanwhile, Park et al. [66] showed that MCP-1 increased the expression of fibronectin and type IV collagen in MCs. Additionally, transfection with mutant MCP-1 and CCR2 siRNA reduced the expression of fibronectin and type IV collagen in MCs cultivated under an HG environment. These findings suggest that MCP-1, which is produced by MCs in HG conditions, may also play an autocrine role in ECM deposition through CCR2.

**Fig. 1:**
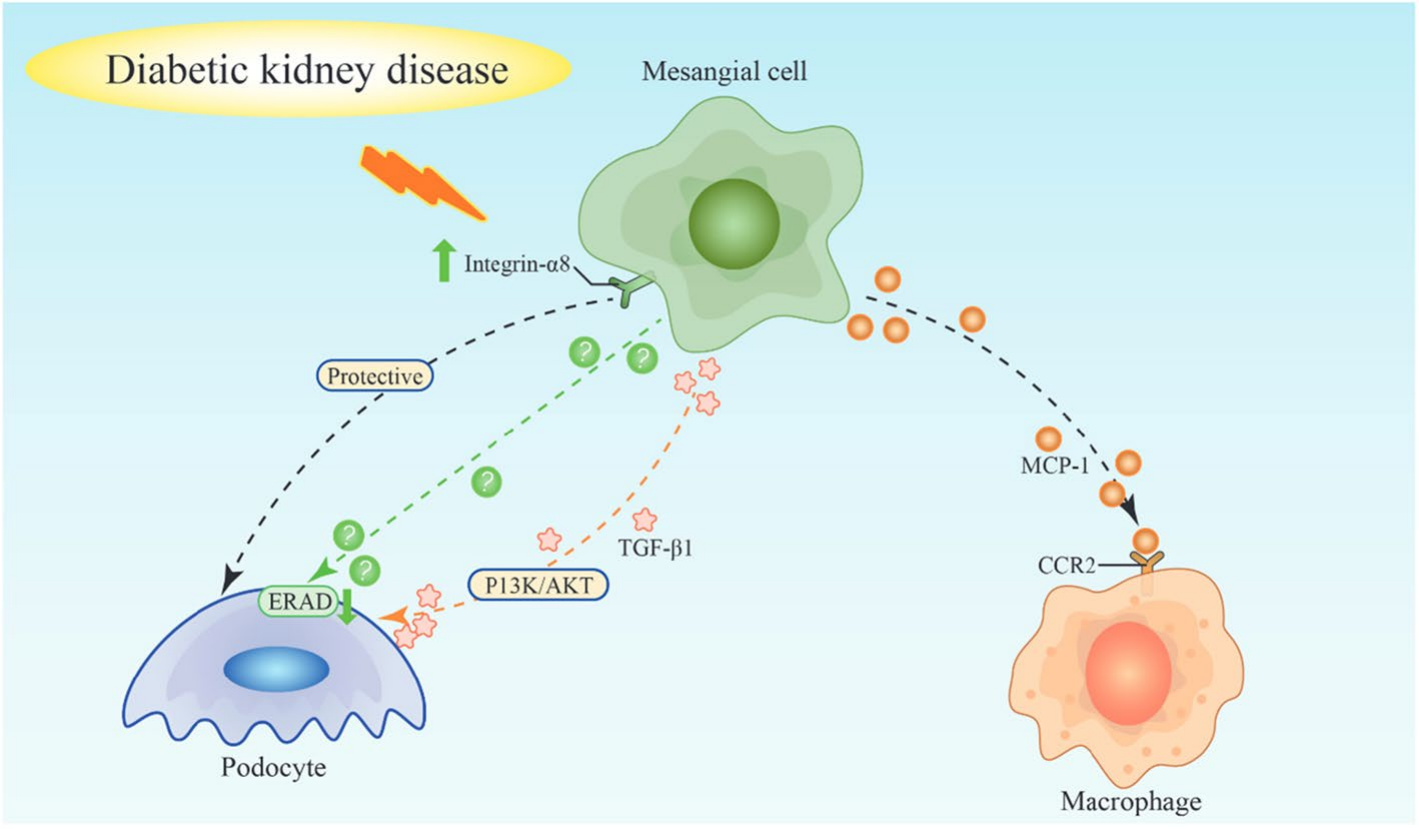
Cellular crosstalk of mesangial cells in diabetic kidney disease. Mesangial cells crosstalk with podocytes via integrin α8, TGF-β1, and ERAD and with macrophages via MCP-1 in diabetic kidney disease. TGF-β1, transforming growth factor-β1; MCP-1, monocyte chemotactic protein 1; CCR2, C-C motif chemokine receptor type 2; ERAD, endoplasmic reticulum-associated degradation

The number of studies on the crosstalk of MCs with other cells in DKD remains limited compared to those on ECs and podocytes. The preceding section describes the crosstalk of MCs with other cells. Overall, regarding DKD, MCs establish crosstalk with podocytes via integrin α8, TGF-β1, and ERAD and with macrophages via MCP-1. The cellular crosstalk of MCs establish with other cells is summarized in Fig. 1.

## Cellular crosstalk of renal TECs

Only one-third of patients with diabetes with microalbuminuria have typical glomerular lesions [67], and renal tubular damage in DKD may precede glomerular damage [68]. Researchers have progressively focused on damage to renal TECs in DKD—biomarkers indicative of proximal tubular damage, including urine kidney injury molecule-1, liver-type fatty acid-binding protein, and neutrophil gelatinase-associated lipocalin (NGAL), are useful for assessing the progression of DN [69, 70]. In the early stages of diabetes, growing renal tubules, including tubular hyperplasia and hypertrophy, are important predictors of increased renal tubular reabsorption, which is closely linked to the upregulation of sodium-glucose-linked cotransporter 2 (SGLT2) [71]. Moreover, increased renal tubular reabsorption reduced the electrolyte load of the dense macula, resulting in the inactivation of the tubuloglomerular feedback mechanism, consequently inducing glomerular hyperfiltration [72]. Daglitazine and other SGLT2 inhibitors have been used in clinical settings to combat this pathological mechanism [73]. As DKD progresses, renal tubules undergo atrophy and fibrosis. Furthermore, a strong correlation has been established between TEC injury and more extensive glomerular injury [74, 75]. Therefore, the crosstalk of TECs in DKD (as described below) is another potential avenue for therapeutic direction.

### Cellular crosstalk of renal TECs with inflammation cells

#### MCP-1

Similar to HG-treated MCs, exposing renal TECs to HG stimulates MCP-1 production [76], a mechanism potentially related to IL-17, IL-18 and ferroptosis [77–79]. Furthermore, Chow et al. [20] reported an increase in MCP-1 production in renal TECs under HG conditions. Using in situ hybridization, WT STZ-treated mice (WT + STZ) were shown to exhibit higher tubular MCP-1 expression levels than their untreated WT counterparts. In diabetic mice, TECs were induced to release MCP-1 at a glucose concentration equivalent to that in the blood [20], further confirming that TECs secrete MCP-1 in DKD. Moreover, MCP-1 can stimulate macrophage recruitment through CCR2. Macrophage aggregation in the kidney is associated with DKD progression, and the underlying mechanism is related to the production of ROS, inflammatory cytokines, and tumor necrosis factor (TNF) [80–83]. Similarly, MCP-1-deficient mice exhibited less glomerular hypertrophy, tubular atrophy, and macrophage infiltration compared to WT diabetic mice [20]. These observations suggest that TECs-derived MCP-1 accelerates the development of DKD by recruiting macrophages.

#### IL-1β

The interleukin family plays a pivotal role in the diagnosis, evaluation, and treatment of DKD. IL-1β is associated with the activation of pyroptosis-related inflammasomes in DKD [84]. Moreover, IL-1β-specific antibodies reduced podocyte loss and macrophage infiltration [85]. IL-1β is also involved in the intercellular crosstalk in DKD. Through flow cytometry and immunofluorescence analyses, Veiras et al. [19] demonstrated elevated levels of IL-1β in diabetic mice, predominantly concentrated in TECs. Cui et al. [86] reported that IL-1β production by TECs in DN was associated with α-kinase 1/NF-κB pathway activation. Under HG conditions, the co-culturing of TECs and macrophages led to an increase in the expression of CD80 (an M1 macrophage marker) in the co-cultured macrophages, whereas that of CD206 (an M2 macrophage marker) was decreased [19]. Furthermore, the authors injected IL-1β-siRNA targeting renal tubules into diabetic mice and found that IL-6 expression in macrophages and mean arterial pressure were lower in experimental mice than in control mice under high-salt dietary conditions. These findings indicate that tubule-derived IL-1β interacts with IL-1 receptor type 1 (IL-1R1) on macrophages in DKD to promote M1 macrophage polarization.

#### miR-199a-5p in extracellular vesicles

Albuminuria, which emerges initially as microalbuminuria and then progresses to macroalbuminuria, is a common DKD symptom [87]. Under this condition, a large amount of albumin is reabsorbed into renal tubules, precipitating tubular damage that consequently triggers kidney inflammation. Jia et al. [88] co-cultured macrophages with human serum albumin (HSA)-treated HK-2 cells in a Transwell co-culture system and reported increased expression of CD86 and TNF-α (M1 macrophage markers) and decreased expression of CD163 (an M2 macrophage marker) in the macrophages, indicating that the cellular crosstalk between TECs and macrophages induces M1 polarization. Transmission electron microscopy and nanoparticle tracking analysis further demonstrated that extracellular vesicles (EVs) from HAS-treated HK-2 cells are taken up by macrophages. This demonstrates that EVs mediate the cellular crosstalk between TECs and macrophages. In addition, miR-199a-5p expression was significantly increased in macrophages exposed to EVs generated from HSA-treated HK-2 cells. The injection of HK-2 cells transfected with si-Rab27a (an exosomal secretion inhibitor) decreased the elevated expression of miR-199a-5p in the macrophages co-cultured with the HAS-treated HK-2 cells. These findings suggest that HSA-treated HK-2 cells can transport miR-199a-5p to macrophages via EVs, leading to M1 polarization [88]. M1 macrophages are proinflammatory cells involved in tissue damage [89]. TargetScan and a luciferase reporter system further identified Klotho as a downstream target of miR-199a-5p-induced macrophage polarization [88].

Additionally, Wu et al. [90] found that miR-199a-5p/Klotho can target Toll-like receptor-4/NF-κB p65/NGAL in MCs under HG conditions to regulate fibrosis and inflammation. The potential for miR-199a-5p in exosomes derived from HSA-treated TECs to facilitate crosstalk with MCs requires verification through subsequent experiments.

#### Hypoxia-inducible factor-1α

Hypoxia-inducible factor-1α (HIF-1α) plays a crucial role in protecting hypoxic cells, enabling their survival under hypoxic conditions [91]. Jiang et al. [92] demonstrated that HIF-1α can upregulate heme oxygenase-1, thereby exerting a protective effect on TECs in DN. Zeng et al. [93] found that TGF-β-activated kinase 1-binding protein 1/NF-kB upregulates HIF-1α in macrophages and promotes glycolysis in DN. Furthermore, Jia et al. [94] found that HSA-treated HK-2 cells promote macrophage glycolysis via EVs, potentially by stabilizing HIF-1α. However, the specific molecules in EVs that mediate HIF-1α stabilization in macrophages remain unknown and require further investigation.

Meanwhile, HIF-1α plays a role in MCs inflammation and fibrosis under HG conditions [95], which may be a potential direction for future research, particularly in exploring TECs crosstalk with MCs.

#### Leucine-rich α-2-glycoprotein 1 in EVs

Leucine-rich α-2-glycoprotein 1 (LRG1) is a secreted glycoprotein that promotes TGF-β-dependent angiogenesis in DKD [96]. Liu et al. reported that urinary LRG1 levels were associated with rapid progression to massive proteinuria and renal impairment in DKD patients [97]. In addition, LRG1 is a marker of renal tubular injury in DKD [98]. Jiang et al. [99] found that TECs from mice with type 2 diabetes mellitus (T2DM) induced by high-density lipoprotein diet secreted LRG1-enriched EVs that targeted TGF-β receptor 1 (TGFβR1), which activated macrophages. Notably, TECs-derived EVs triggered macrophages to release their EVs (tumor necrosis factor-related apoptosis-inducing ligand-enriched), targeting death receptor 5 and consequently inducing apoptosis in TECs.

Additionally, according to Hong et al. [100], TECs-derived LRG1 can interact with fibroblasts, stimulate the TGF-β/Smad3 pathway, and promote fibrosis in a renal fibrosis model. However, further studies are required to determine whether LRG1-mediated cellular crosstalk exists between TECs and fibroblasts in DKD.

#### Delta-like protein 4 in exosomes

Delta-like protein 4 (Dll4) is an important regulator of the Notch signaling pathway [101] and a key molecule in diabetic vasculopathy [102]. Under HG conditions, blocking Dll4/Notch inhibited neovascularization [103]. Zhu et al. [104] reported high serum Dll4 levels as a biomarker for diabetic retinopathy. Additionally, in DKD, the Notch pathway activation promoted macrophage polarization [105]. Liu et al. [106] found that exosomes secreted by HG-treated TECs promoted M1 polarization and identified another crosstalk molecule, Dll4, in exosomes. Dll4 in exosomes secreted from HG-treated TECs can facilitate crosstalk with macrophages and promote M1 polarization—Dll4 expression was regulated by Epsin1. The potential role of Dll4 in DKD remains unclear and requires further in-depth exploration.

#### C-C chemokine ligand 21/C-C motif chemokine receptor type 7

T, B, and dendritic cells are chemotactically affected by the C-C chemokine ligand 21 (CCL21; also known as secondary lymphoid tissue chemokine)/C-C motif chemokine receptor type 7 [CCR7]) axis, which plays a role in the immune response [107, 108]. Moreover, CCL21/CCR7 can recruit T cells to pancreatic islets and play an important role in inflammatory response in type 1 diabetes mellitus [109]. Feng et al. [110] observed increased urinary CCL21 mRNA expression levels in urinary small EVs secreted by renal tubules in patients with DN than in those with diabetes mellitus (DM). Additionally, the expression of CCL21 in the kidney tissue of patients with DN was localized in the tubulointerstitium, further supporting the notion that renal TECs produce CCL21. CCL21 recruits T lymphocytes via CCR7 [111]. Feng et al. [110] reported the colocalization of CCL21 and CD3 + T cells in the renal tubules of DN kidney tissue, suggesting that the recruitment of CD3 + T cells in DN is related to CCL21 secreted by the renal tubules. Additionally, Moon et al. [112] reported that in the kidney tissues of diabetic mice, CD3 + T cells produced interferon-γ and TNF-α, which play a proinflammatory role in DN.

In addition, it was reported that CCL21 activation of CCR7 on MCs enhances cell adhesion and thus increases the formation of cell-cell contacts [113]. Whether CCL21 can mediate crosstalk between TECs and MCs in DKD is a promising research direction.

Overall, these studies emphasize that inflammatory pathways play a significant role in DKD. Hence, therapies targeting different inflammatory mediators are promising research avenues for DKD. Further information regarding potential therapeutics that target inflammatory pathways in DKD is summarized in Table 1.

**Table 1:** Potential therapeutic agents targeting inflammatory mechanisms in DKD

Drug	Mechanism	Clinical trial result	Reference
Pentoxifylline (PTF)	Nonspecific inhibition of phosphodiesterase	Addition of PTF to renin-angiotensin system (RAS) inhibitors resulted in a smaller decrease in the estimated glomerular filtration rate (eGFR) and a greater reduction of residual albuminuria than with RAS inhibitors alone.	[114]
Bardoxolone methyl	Inhibition of proinflammatory signals and oxidative stress	Bardoxolone methyl might delay the onset of ESRD in patients with T2DM and stage 4 chronic kidney disease.	[115]
Selonsertib	Small-molecule apoptosis-signal-regulating kinase 1 inhibitor	Selonsertib might slow DKD progression.	[116]
Baricitinib	JAK1 and JAK2 inhibitors	Baricitinib decreased albuminuria in participants with T2DM and DKD.	[117]
CCX140-B	CCR2 inhibitors	CCX140-B reduced proteinuria in T2DM patients with nephropathy.	[118]
ASP8232	Vascular adhesion protein-1 inhibitor	ASP8232 was effective in reducing albuminuria in patients with diabetic kidney disease and was safe and well tolerated.	[119]

### Cellular crosstalk of renal TECs with glomerular cells

#### Cellular crosstalk of renal TECs with podocytes

##### Gremlin

Gremlin is a member of the bone morphogenetic protein antagonist family [120]. By transfecting diabetic mice with gremlin siRNA plasmids, Zhang et al. [121] demonstrated the protective effect of gremlin inhibition on renal function. Furthermore, Roxburgh et al. [122] induced diabetes in *gremlin1* heterozygous-knockout mice and observed reduced GBM thickening and ECM deposition relative to those in WT diabetic mice. These findings indicate that gremlin plays a role in promoting the occurrence and development of DKD.

MCs and podocytes can express gremlin under HG conditions [123, 124]. Gremlin-treated podocytes exhibited decreased nephrin and synaptopodin expression levels, which may be attributed to the TGF-β/Smad signaling pathway [124]. Moreover, HG-induced gremlin expression in MCs was associated with ECM deposition via the extracellular signal-regulated kinase 1/2 pathway [125]. However, Dolan et al. [126] reported that gremlin was predominantly expressed in regions of renal tubulointerstitial fibrosis in DN and was only occasionally expressed in the glomerular area. This discrepancy in expression patterns across locations implies that gremlin may undergo intercellular crosstalk in DKD. Marchant et al. [127] established an STZ-induced DN mouse model using transgenic mice overexpressing tubule-specific gremlin. Compared to the WT diabetic mice, the transgenic DN mice exhibited more severe tubulointerstitial damage, concomitant with substantially decreased podocin expression, foot process disappearance, decreased podocyte number (marked by WT-1), and inflammatory cell infiltration. These findings indicate that tubular gremlin overexpression in DKD induces cellular crosstalk that damages podocytes. Additionally, gremlin overexpression in renal tubules resulted in more severe inflammatory cell infiltration, which may be attributed to increased MCP-1 expression [128].

Gremlin may exert an autocrine effect—it can promote renal fibrosis and inflammatory responses by binding to vascular endothelial growth factor receptor-2 (VEGFR-2) [128, 129]. Lavoz et al. [130] cultured renal TECs in the presence of gremlin and measured the expression levels of Jagged-1 and Notch-1. They found that gremlin activated the Notch pathway in renal TECs; however, this activation was counteracted by VEGFR-2 inhibitors. These results led to the conclusion that gremlin binds to VEGFR-2 and activates the Notch pathway in renal TECs, highlighting their autocrine role in TECs.

##### Bcl-2 interacting mediator of cell death

Bcl-2 interacting mediator of cell death (Bim), a Bcl-2 homology domain 3-only protein belonging to the Bcl-2 family, induces apoptosis [131]. Zhang et al. [132] reported that HG-induced apoptosis was initiated following upregulated Bim expression in proximal TECs and that Bim silencing protected TECs from HG-mediated apoptosis. Furthermore, calcium dobesilate, prostaglandin E1 and salidroside delayed DKD progression by inhibiting Bim, thereby attenuating the apoptosis of proximal renal TECs [133–135]. Therefore, targeting Bim is a promising therapeutic strategy for DKD.

Xu et al. [136] co-cultured HG-treated proximal TECs (PTECs) with podocytes and found that exposure to HG resulted in increased Bim expression in PTECs, decreased synaptophysin expression, and rearrangement of F-actin in podocytes. These findings suggest that HG-treated PTECs interact with podocytes and induce damage. In addition, transfection of PTECs with Lenti-Bim-shRNA reversed cytoskeletal disturbances in podocytes co-cultured with HG-treated PTECs, indicating that Bim is an essential molecule in the crosstalk between HG-treated PTECs and podocytes.

Moreover, nuclear factor of activated T cells 2 (NFAT2) overexpression counteracted the orderly arrangement of the cytoskeleton resulted by the inhibition of Bim expression, suggesting that NFAT2 is a downstream target of Bim [136]. Further, Li et al. demonstrated that NFAT2 could target Bax to induce podocyte apoptosis under HG conditions [137]. Overall, these findings indicate that under HG conditions, TECs induce cytoskeletal abnormalities in podocytes via the Bim/NFAT2 pathway.

##### Sirtuin 1

Sirtuin1 (Sirt1) is a nicotinamide adenine dinucleotide-dependent deacetylase that targets NF-κB, p53, TGF-β1, and p66Shc, among others, to exert its anti-inflammatory, anti-apoptotic, and anti-fibrotic effects; it also protects mitochondrial functions in DN [138]. Hasegawa et al. [139] established a proximal tubule-specific Sirt1 transgenic diabetic mouse model (TG + STZ). Compared to WT + STZ mice, TG + STZ mice showed decreased urinary albumin excretion and increased dense pore density, suggesting that Sirt1 plays a renoprotective role in DKD.

*Claudin-1* was identified as a key gene through DNA microarray analysis of glomeruli of WT + STZ and TG + STZ mice [139]. Its overexpression reduced nephrin and podocin expression, destabilized the podocyte slit diaphragm, and induced podocyte damage [140]. Furthermore, in WT + STZ mice, Claudin-1 was mainly localized in parietal epithelial cells (PECs) and podocytes, exhibiting increased expression [139]. Sirt1 expression was also decreased in the renal tubules during the early stage of diabetes. Moreover, the TG + STZ group exhibited a reversal of the increased Claudin-1 expression observed in podocytes and PECs in the WT + STZ group. This study suggests that Sirt1, localized in the renal tubules, is involved in downregulating Claudin-1 expression in podocytes and PECs in DN. The potential mechanism underlying this process is the induction of the methylation of CpG islands in *Claudin-1*. In DKD, reduced Sirt1 expression in TECs attenuated the inhibitory effect of Claudin-1 in podocytes, leading to podocyte damage. Furthermore, Itaru et al. [141] reported that nicotinamide mononucleotide (NMN) treatment reduced urinary albumin excretion and foot process disappearance in DN mice through Sirt1 upregulation and NAD + salvage pathway activation. Therefore, by injecting exogenous NMN labeled with N-methylanthraniloyl into diabetic mice, the potential for NMN to induce crosstalk between renal TECs and podocytes in DKD was validated [139]. In conclusion, NMN is an important mediator of TECs regulation of Claudin-1 expression in podocytes and PECs via Sirt1.

#### Cellular crosstalk of renal TECs with ECs

##### Angiopoietin

Angiopoietin 1 (Ang1) exerts a protective effect by stabilizing blood vessel walls, whereas angiopoietin 2 (Ang2) can disrupt capillary integrity. Jiang et al. [15] have demonstrated the involvement of angiopoietins in the crosstalk between podocytes and ECs. Other studies have also provided evidence for the involvement of angiopoietins in the crosstalk between renal TECs and ECs. Via immunohistochemical staining, Rizkalla et al. [142] found that Ang1 was primarily distributed in renal TECs, whereas Ang2 and Tie, a co-receptor for Ang1/2, were located in the ECs of diabetic mice. Additionally, Ang2 expression increased as diabetes progressed, whereas Ang1 expression increased at week 4 and decreased at week 8. Furthermore, increases in Ang1 and Ang2 levels were disproportionate, and the Ang2/Ang1 ratio increased with disease progression. Overall, the differential expression of Ang2 and Ang1 plays a role in the pathogenesis of DKD, with Ang1 deficiency reducing the protective effects of TECs on ECs in DKD.

#### Cellular crosstalk of renal TECs with MCs

##### miR-92a-1-5p in exosomes

Using a co-culture system, Tsai et al. [143] observed that exosomes produced by HG-treated HK-2 cells promoted ER stress and myofibroblast transdifferentiation (MFT) in MCs. Furthermore, miR-92a-1-5p expression was elevated in exosomes, and mimics promoted MFT in MCs. These findings suggest that TECs-derived exosomes induce MFT in MCs via miR-92a-1-5p under HG conditions. Additionally, MFT is involved in renal fibrosis [144], and RCN3 was identified as the downstream target of miR-92a-1-5p. High levels of urinary exosomal miR-92a-1-5p were associated with low eGFR levels in patients with T2DM [143].

### Cellular crosstalk of renal TECs with fibroblasts

#### miR-196b-5p in EVs

Increased RAS activity, including aldosterone synthesis, has been associated with DM [145]. Aldosterone is a mineralocorticoid hormone that promotes inflammatory cell infiltration, collagen deposition, and renal tubular dilatation [146]. Hu et al. [147], demonstrated that TECs from aldosterone-treated diabetic mice showed decreased E-cadherin expression and increased NGAL expression. Furthermore, the expression levels of fibronectin, α-smooth muscle actin, and collagen type I α1 chain were increased in aldosterone-treated diabetic mice. However, the expression of mineralocorticoid receptors was relatively low in fibroblasts. These findings suggest that aldosterone does not act directly on fibroblasts. A co-culture system of PTECs with fibroblasts showed that PTECs can establish a crosstalk with fibroblasts through EVs. Furthermore, miR-196b-5p was found to be abundant in these EVs, as shown by the miRNA array analysis of the EVs produced from PTECs. Fibroblasts transfected with miR-196b-5p mimics promoted ECM production, further confirming that PTEC promotes DKD fibrosis by establishing crosstalk with fibroblasts via miR-196b-5p. The action mechanism of miR-196b-5p in fibroblasts may be related to the STAT3/SOCO2 pathway, as the miR-196b-5p mimic also enhances STAT3 phosphorylation and inhibits SOCS2 expression in fibroblasts [147]. Effective antifibrotic treatment during the early phases of renal fibrosis is an essential strategy for reducing the progression of DKD. The novel drugs targeting renal fibrosis in DKD are summarized in Table 2.

**Table 2:** Novel drugs targeting renal fibrosis in DKD

Drug	Randomized controlled trial (NCT)	Stage	Current clinical trial result
Pirfenidone	NCT00063583	completed	[148]
	NCT02689778	completed	unknow
Finerenone	NCT02545049	completed	[149, 150]
	NCT02540993	completed	[151]
	NCT01874431	completed	[152, 153]
	NCT01968668	completed	[153]
Paricalcitol	NCT00421733	completed	[154]

**Fig. 2:**
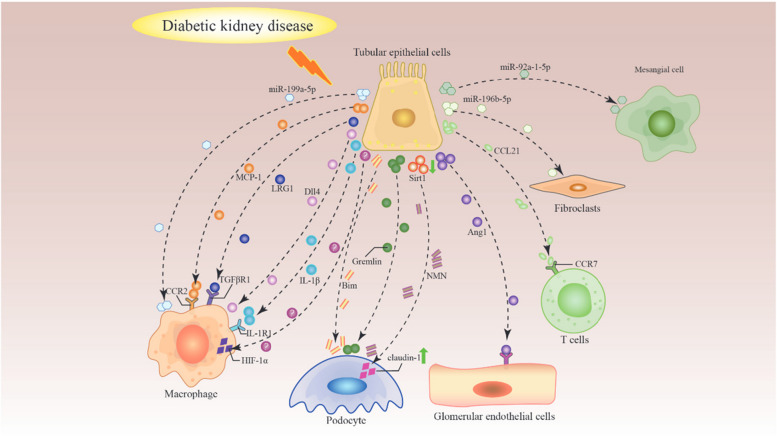
Cellular crosstalk of renal tubular epithelial cells in diabetic kidney disease. In diabetic kidney disease, tubular epithelial cells establish crosstalk with podocytes via gremlin, Bim, and Sirt-1/NMN and with endothelial cells via Ang1. Additionally, tubular epithelial cells recruit/activate macrophages via MCP-1, IL-1β, Dll4, LRG1, HIF-1α, and miR-199a-5p and recruit T cells via CCL21. Tubular epithelial cells also exert crosstalk effects on fibroblasts and mesangial cells via miR-196b-5p and miR-92a-1-5p, respectively. IL-1β, interleukin-1β; IL-1R1, interleukin-1 receptor type 1; CCL21, C-C chemokine ligand 21; CCR7, C-C motif chemokine receptor type 7; NMN, nicotinamide mononucleotide; Sirt1, Sirtuin 1; MCP-1, monocyte chemotactic protein 1; CCR2, C-C motif chemokine receptor type 2; Bim, B cell lymphoma-2 interacting mediator of cell death; Ang1, angiopoietin 1; Dll4, delta-like protein 4; LRG1, leucine-rich α-2-glycoprotein 1; TGFβR1, transforming growth factor-β receptor 1; HIF-1α, hypoxia-inducible factor-1α

In line with the previous discussions above, TECs can expand proinflammatory and profibrotic effects in DKD by establishing crosstalk with other cells. The cellular crosstalk of TECs is summarized in Fig. 2.

## Conclusions

**Fig. 3:**
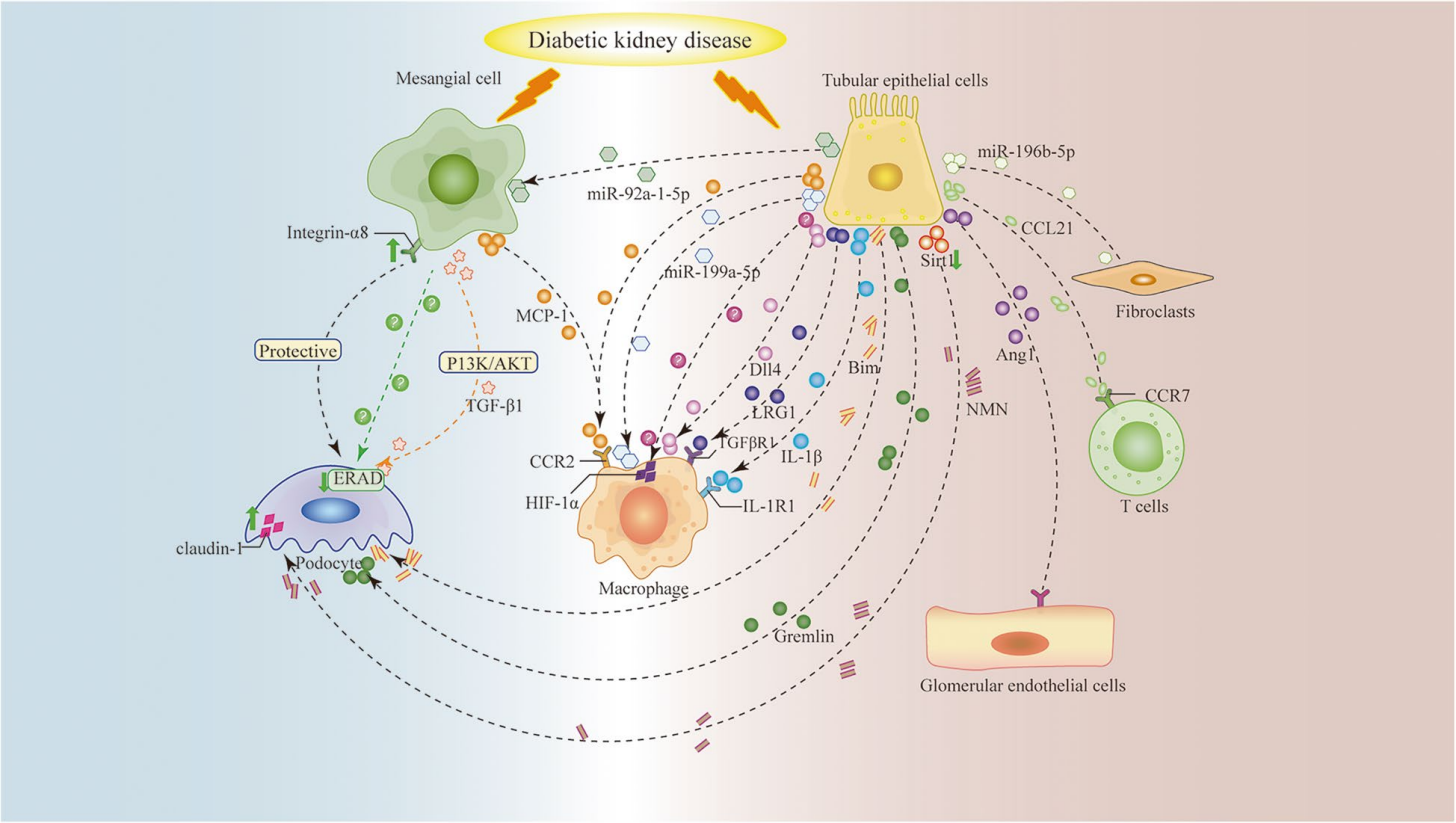
Cellular crosstalk of mesangial cells and renal tubular epithelial cells. A summary about the crosstalk of tubular epithelial cells and mesangial cells with other cells in DKD. TGF-β1, transforming growth factor-β1; MCP-1, monocyte chemotactic protein 1; CCR2, C-C motif chemokine receptor type 2; ERAD, endoplasmic reticulum-associated degradation; IL-1β, interleukin-1β; IL-1R1, interleukin-1 receptor type 1; CCL21, C-C chemokine ligand 21; CCR7, C-C motif chemokine receptor type 7; NMN, nicotinamide mononucleotide; Sirt1, Sirtuin 1; Bim, B cell lymphoma-2 interacting mediator of cell death; Ang1, angiopoietin 1; Dll4, delta-like protein 4; LRG1, leucine-rich α-2-glycoprotein 1; TGFβR1, transforming growth factor-β receptor 1; HIF-1α, hypoxia-inducible factor-1α

To gain a comprehensive understanding of the mechanisms underlying cellular crosstalk in DKD, this review explores the cellular crosstalk of TECs and MCs with other cells (which has been summarized in Fig. 3; Table 3). Although several mechanisms underlying cellular crosstalk in DKD remain largely unknown and require further investigation, the CellChat analysis program created by Jin et al. may offer guidance for further investigation into cellular crosstalk in DKD [155]. The main conclusions drawn are listed below:

**Table 3:** Cellular crosstalk of renal tubular epithelial cells and mesangial cells

	Factor/ Signal pathway	Crosstalk cell	Effect	Reference
Tubular epithelial cells	Gremlin	Podocytes	pathogenic	[127]
	Bim		pathogenic	[136]
	Sirt1/NMN		protective	[139]
	Ang1	Endothelial cells	protective	[142]
	MCP-1	Macrophages	pathogenic	[20]
	miR-199a-5p in EVs		pathogenic	[88]
	IL-1β		pathogenic	[19]
	Dll4 in exosomes		pathogenic	[106]
	LRG1 in EVs		pathogenic	[99]
	HIF-1α		pathogenic	[94]
	CCL21	T cells	pathogenic	[110]
	miR-196b-5p in EVs	Fibroblasts	pathogenic	[147]
	miR-92a-1-5p in exosomes	Mesangial cells	pathogenic	[143]
Mesangial cells	Integrin α8	Podocytes	protective	[18, 44, 45]
	TGF-β1 in exosomes		pathogenic	[36]
	ERAD		protective	[40]
	MCP-1	Macrophages	pathogenic	[60, 64, 65]

1. In DKD, MCs establish crosstalk with podocytes via integrin α8, TGF-β1, and ERAD and with macrophages via MCP-1.
2. In DKD, TECs establish crosstalk with podocytes via gremlin, Bim, and Sirt-1/NMN and with ECs via Ang1. Additionally, TECs recruit/activate macrophages via MCP-1, IL-1β, Dll4, LRG1, HIF-1α, and miR-199a-5p and recruit T cells via CCL21. TECs also exert crosstalk effects on fibroblasts and MCs via miR-196b-5p and miR-92a-1-5p, respectively.
3. Among the various molecules involved in the crosstalk of TECs and MCs, pathogenic factors include TGF-β1, MCP-1, gremlin, miR-196b-5p, Bim, IL-1β, CCL21, miR-199a-5p, Dll4, LRG1, HIF-1α, and miR-92a-1-5p, whereas protective factors include integrin α8, ERAD, Ang1, and Sirt1.

The following are unresolved/unexplored issues in the field:

1. Few studies have been conducted on cellular crosstalk between TECs and MCs. Only Tsai et al. [143] have reported miR-92a-1-5p crosstalk between TECs and MCs. Therefore, further research is required to gain a comprehensive understanding of this unexplored subject.
2. Inflammatory cell recruitment accelerates the advancement of DKD. MCP-1, miR-199a-5p, IL-1β, Dll4, LRG1, HIF-1α, and CCL21 are implicated in the aggregation of inflammatory cells in DKD; however, additional inflammatory mechanisms require investigation.
3. In DKD, intercellular crosstalk is complex, and most studies have only elucidated potential molecules involved in cellular crosstalk. Thus, more studies are required to elucidate the underlying complex mechanisms.

## Data Availability

Not applicable.

## Abbreviations

AGEs: Advanced glycation end products
Ang1: Angiopoietin 1
Ang2: Angiopoietin 2
Bax: B-cell lymphoma 2 associated protein X
Bcl-2: B-cell lymphoma 2
Bim: Bcl-2 interacting mediator of cell death
CCL21: C-C chemokine ligand 21
CCR2: C-C chemokine receptor type 2
CCR7: C-C motif chemokine receptor type 7
CHOP: C/EBP homologous protein
DKD: Diabetic kidney disease
Dll4: Delta-like protein 4
DM: Diabetes mellitus
DN: Diabetic nephropathy
ECM: Extracellular matrix
ECs: Endothelial cells
eGFR: Estimated glomerular filtration rate
ER: Endoplasmic reticulum
ERAD: Endoplasmic reticulum-associated degradation
ESRD: End-stage renal disease
EVs: Extracellular vesicles
GBM: Glomerular basement membrane
GRP: Glucose-regulated protein
HG: High glucose
HIF-1α: Hypoxia-inducible factor-1α
HSA: Human serum albumin
IL: Interleukin
IL-1R1: IL-1 receptor type 1
JAK: Janus kinase
LRG1: Leucine-rich α-2-glycoprotein 1
MCP-1: Monocyte chemoattractant protein-1
MCs: Mesangial cells
MFT: Myofibroblast transdifferentiation
NFAT2: Nuclear factor of activated T cells 2
NF-κB: Nuclear factor κB
NGAL: Neutrophil gelatinase-associated lipocalin
NMN: Nicotinamide mononucleotide
PECs: Parietal epithelial cells
PI3K: Phosphatidylinositol 3-kinase
PTECs: Proximal tubular epithelial cells
PTF: Pentoxifylline
RAS: Renin-angiotensin system
ROS: Reactive oxygen species
SGLT2: Sodium-glucose-linked cotransporter 2
Sirt1: Sirtuin1
STAT: Signal transducer and activator of transcription
STZ: Streptozotocin
T2DM: Type 2 diabetes mellitus
TECs: Tubular epithelial cells
TGF-β: Transforming growth factor-β
TGFβR1: Transforming growth factor-β receptor 1
TNF: Tumor necrosis factor
TRB3: Tribbles homolog 3
UPR: Unfolded protein response
VEGFR-2: Vascular endothelial growth factor receptor-2
WT: Wild-type
